# Oral Favipiravir Exposure and Pharmacodynamic Effects in Adult Outpatients With Acute Influenza

**DOI:** 10.1093/infdis/jiad409

**Published:** 2023-09-22

**Authors:** Frederick G Hayden, Robert P Lenk, Carol Epstein, Lih Lisa Kang

**Affiliations:** Division of Infectious Diseases and International Health, Department of Medicine, University of Virginia School of Medicine, Charlottesville, Virginia, USA; MediVector, Inc, Boston, Massachusetts, USA; MediVector, Inc, Boston, Massachusetts, USA; MediVector, Inc, Boston, Massachusetts, USA

**Keywords:** favipiravir, influenza, pharmacokinetics, pharmacodynamics, antiviral effects

## Abstract

**Background:**

The pharmacokinetics of oral favipiravir and the relationships of plasma concentrations to antiviral effects are incompletely studied in influenza.

**Methods:**

Serial plasma samples were collected from adults with uncomplicated influenza who were randomized to favipiravir (1800 mg twice a day on day 1, 800 mg twice a day on days 2 to 5; n = 827) or placebo (n = 419) in 2 phase 3 trials. Post hoc analyses assessed the frequency of reaching an average minimum concentration (C_min_) ≥20 µg/mL, its association with antiviral efficacy, and factors associated with reduced favipiravir exposure.

**Results:**

Wide interindividual variability existed in favipiravir concentrations, and this regimen failed to reach an average C_min_>20 µg/mL in 41%–43% of participants. Those attaining this threshold showed greater reductions in nasopharyngeal infectious virus titers on treatment days 2 and 3 and lower viral titer area under the curve compared to those who did not. Those with average C_min_ <20 µg/mL had over 2-fold higher mean ratios of the metabolite T-705M1 to favipiravir, consistent with greater metabolism, and were more likely to weigh >80 kg (61.5%–64%).

**Conclusions:**

Higher favipiravir levels with average C_min_>20 µg/mL were associated with larger antiviral effects and more rapid illness alleviation compared to placebo and to favipiravir recipients with lower average C_min_ values in uncomplicated influenza.

***Clinical Trials Registration***. NCT1068912 and NCT01728753.

## Teaser

In influenza treatment, oral favipiravir failed to consistently reach target plasma concentrations and showed wide interindividual variability in concentrations associated with differences in favipiravir's metabolism and body weight. Higher oral doses and in seriously ill patients intravenous dosing require study.

Favipiravir (T-705) is a nucleic acid analog prodrug that was initially developed as a treatment for influenza and has been subsequently tested as a potential therapy for a number of human infections caused by RNA viruses, including ebolavirus, severe acute respiratory syndrome coronavirus 2 (SARS-CoV-2), and rabies [1–6]. The drug has been under study for influenza for approximately 2 decades and was approved in Japan in 2014 for treatment of novel or reemerging influenza virus infections in which other antiviral drugs are ineffective. The 50% effective concentrations (EC_50_s) for inhibition of influenza A and B viruses, including those resistant to other influenza antivirals, ranges generally from 0.014 to 0.55 μg/mL in MDCK cells [1], although concentrations up to 3.53 µg/mL (22.5 uM) are required for some influenza strains [2, 7]. Favipiravir-ribofuranosyl-5'-triphosphate (RTP) mimics a purine analog [8–10] and may cause chain termination or be incorporated into RNA chains and act as a viral mutagen [10–12].

Oral favipiravir has complex human pharmacokinetics (PK), and optimal dose regimens remain uncertain [13]. Although it appears to be efficiently absorbed after oral administration, favipiravir is both a substrate for and inhibitor of aldehyde oxidase (AO), such that loading dose regimens have been used in most clinical studies. The primary route of metabolism is via enzymes in AO/xanthine oxidase family that convert favipiravir to the inactive metabolite T-705M1 which is excreted primarily in the urine. Of note, AO is present in the liver, lungs, small and large intestine, kidney, prostate, and adrenal glands [14].

In a phase 2 study in uncomplicated influenza (US204; NCT1068912), participants with the highest quartile minimum plasma concentration (C_min_) of favipiravir >19.5 µg/mL after the first 24 hours of dosing demonstrated the greatest antiviral efficacy and the shortest time to alleviation of influenza symptoms compared to placebo (Carol Epstein, unpublished data) [15]. Another phase 2 study (US213; NCT01728753) comparing twice a day (1800 mg twice a day for 2 doses, followed by 800 mg twice a day) and 3 times a day (2400 mg once, followed by 600 mg twice on first day, and then 3 times a day) dosing regimens found that the twice a day regimen was more reliable in achieving C_min_ ≥20 µg/mL 24 hours after starting administration and was also associated with significant clinical and antiviral efficacy compared to placebo, whereas the 3 times a day regimen was less effective (Carol Epstein, unpublished data) [15].

Two phase 3 trials in adults with uncomplicated influenza testing the twice a day dosing regimen demonstrated significant antiviral effects but inconsistent differences in reducing median times to illness alleviation compared to placebo (14.4 vs 6.1 hours) [16]. A post hoc analysis of recipients who had average C_min_ ≥20 µg/mL over the 5 treatment days found that the median time to illness alleviation for favipiravir recipients was 83.3 hours (95% confidence interval [CI], 71.8–95.5 hours; *P* = .003 vs placebo) compared to 95.7 hours (95% CI, 77.1–101.1 hours) for those with lower average C_min_ in US316 [16], suggesting that those with higher exposures had greater reductions in time to illness alleviation. In this article we describe additional post hoc analyses undertaken to assess the relationships between favipiravir exposure and the key virologic end points in the 2 trials, as well as the factors affecting plasma favipiravir concentrations.

## METHODS

### Overview

As detailed previously [16], participants received 1800 mg favipiravir twice a day during the first 24 hours, and then 800 mg twice a day over the subsequent 4 days, or matching placebo tablets. Participants filled out a diary 3 times a day for 21 days to record body temperature, symptoms, adverse events, and timing of study drug dosing, and were seen daily for study days 1 (baseline) to 5 for clinical monitoring and collection of nasopharyngeal swabs for infectious virus quantitation.

### Pharmacokinetic Sampling

During the first 5 days, blood samples for measuring concentrations of favipiravir and its major metabolite T-705M1 were taken within 30 minutes prior to dosing to measure trough concentrations (C_min_) and 45 to 75 minutes following dosing to capture peak concentrations (C_max_) on days 1, 2, 3, 4, and 5. Thus, half the doses were taken under observation in the clinic.

Plasma samples were kept frozen at −20°C or colder prior to analysis. The plasma concentrations of favipiravir and its major metabolite T-705M1 were performed by PPD Bioanalytical Services and Clinical Pharmacology, Richmond, VA by high-performance liquid chromatography (HPLC). The lower limit of quantification (LLOQ) in the assay was 0.020 µg/mL for both favipiravir and T-705M1.

The PK population included all participants who received favipiravir and who had at least 1 plasma sample after baseline with a detectable favipiravir level. Those analyzed in this report are those in the PK population who had virologically confirmed influenza infection.

### Clinical and Virologic End Points

The primary clinical end point was the time in hours from the start of study drug administration until illness alleviation, defined as absence or mild severity of all 6 primary influenza symptoms for at least 21.5 hours. The analyses were performed on participants in the intent-to-treat infected (ITTI) groups, which included all who received at least 1 dose of study drug and were subsequently determined to be influenza virus-positive by either reverse transcription polymerase chain reaction (RT-PCR) or culture on study day 1. The time to symptom alleviation was analyzed by the Peto-Peto-Prentice test.

The main virology objective was to evaluate the antiviral effects of favipiravir compared with placebo in nasopharyngeal swab samples during acute influenza. The specific end points included change in viral titers measured by median tissue culture infectious dose (TCID_50_) in the nasopharyngeal swab samples at visits 2, 3, 4, and 5 compared to baseline; viral titer (TCID_50_) area-under-the-curve (AUC) values through visit 5 using the trapezoidal method; and the time to cessation of infectious virus detection. AUCs were not calculated for a participant if any values were missing at baseline, day 3, or day 5. Day 2 and day 4 values, if available, were used in the calculation of AUCs. ANCOVA of the AUCs of TCID_50_ (with baseline as a covariate) was carried out for comparison between the placebo and favipiravir groups.

### Pharmacokinetic-Pharmacodynamic Analyses

Favipiravir exposure measures were analyzed with regard to the pharmacodynamic end point of antiviral efficacy using the above virologic outcomes, as well as the primary clinical end point of illness alleviation. Exposure to favipiravir in participants was assessed by measuring maximum (C_max_) and minimum (C_min_) plasma concentrations at intervals (as given above). The average C_min_ for days 2–5 was calculated for each favipiravir recipient. In addition, the proportions who did or did not achieve the target C_min_ value of ≥20 µg/mL were determined for each sampling time point.

Descriptive statistics for plasma concentrations of favipiravir and T-705M1 were performed for each day and time point. Plasma concentrations of favipiravir that were below the limit of quantification were set to 0. To assess the relationship between plasma concentrations of favipiravir and T-705M1, we examined the correlations between favipiravir C_max_ and C_min_ and the corresponding T-705M1 concentration at each time point. We then assessed the effect of C_min_ on the ratio of T-705M1 to favipiravir by comparing participants with average C_min_ <20 µg/mL to those with average C_min_ ≥20 µg/mL using a mixed model for repeated measures.

The effect of favipiravir exposure on infectious virus titers was tested between C_min_ status compared to placebo and between C_min_ <20 µg/mL versus C_min_ ≥20 µg/mL at each time point with a generalized linear model. Because those weighing >80 kg showed no benefit in illness alleviation from favipiravir treatment [16], we also examined the proportions of participants with low average C_min_ or day 1 C_max_ values for those weighing <80 kg compared to ≥80 kg, as well as the relationship of body weight to viral titer AUC values.

## RESULTS

### Study Populations

Among ITTI participants (301 favipiravir, 322 placebo) in US316, evaluable PK sampling data were available for 294 (97.7%) favipiravir recipients. Among the ITTI population (526 favipiravir, 169 placebo) in US317, adequate PK data were available for 510 (97.0%). Seven participants in US316 and 15 in US317 without PK samples were excluded from analysis.

The enrollment characteristics of the favipiravir and placebo groups in the ITTI populations with adequate PK sampling data were comparable in the 2 trials (Table 1). Most participants were infected with influenza type A, with A/H3N2 being the predominant subtype. Baseline infectious virus titers were similar in favipiravir and placebo recipients within each trial, but mean titers were 0.6 log_10_ TCID_50_/mL higher in the favipiravir group of US317 compared to US316.

**Table 1.** Enrollment Demographic and Illness Characteristics of the Participants in the ITTI Populations With Adequate Pharmacokinetics Sampling Data for Analysis

Characteristic	US316		US317	
	Placebo (n = 322)	Favipiravir (n = 301)	Placebo (n = 169)	Favipiravir (n = 526)
Age, y, mean (SD)	41.3 (14.8)	41.3 (14.2)	39.3 (14.19)	40.1 (13.61)
Female sex, No. (%)	192 (59.6)	176 (58.5)	87 (51.5)	305 (58.0)
Race, No. (%)				
African American	54 (16.8)	40 (13.3)	14 (8.3)	64 (12.2)
American Indian or Alaska Native	0 (0.0)	2 (0.7)	8 (4.7)	17 (3.2)
Asian	14 (4.3)	5 (1.7)	0 (0.0)	9 (1.7)
Native Hawaiian or Other Pacific Islander	0 (0.0)	0 (0.0)	0 (0.0)	0 (0.0)
White	241 (74.8)	246 (81.7)	118 (69.8)	348 (66.2)
Multiple	0 (0.0)	0 (0.0)	2 (1.2)	4 (0.8)
Other	13 (4.0)	10 (3.4)	27 (16.0)	84 (16.0)
Ethnicity, No. (%)				
Hispanic or Latino	46 (14.3)	46 (15.3)	85 (50.3)	250 (47.5)
Body mass index, kg/m^2^, mean (SD)	29.0 (7.0)	29.0 (7.4)	28.6 (6.2)	29.0 (6.4)
Weight, kg, mean (SD)	81.8 (20.8)	83.2 (22.4)	80.4 (20.7)	81.5 (20.8)
Influenza vaccine in current season, No. (%)	68 (21.1)	64 (21.3)	20 (11.8)	45 (8.6)
Time from symptom onset to first dose, h, mean (SD)	29.9 (10.6)	29.2 (10.5)	30.2 (10.2)	29.3 (10.7)
Time from symptom onset to first dose <24 hours, No. (%)	96 (29.8)	90 (29.9)	42 (24.9)	164 (31.2)
Temperature, °C, mean (SD)	38.0 (0.9)	37.9 (0.8)	37.8 (0.8)	37.9 (0.8)
Baseline symptom score ≥15, No. (%)	60 (20.1)	67 (23.5)	41 (26.3)	132 (26.5)
Viral titer, log_10_ TCID_50_/mL, mean (SD)	3.0 (1.8) [n = 321]	2.8 (1.7) [n = 301]	3.3 (2.0) [n = 169]	3.4 (1.9) [n = 526]
Viral RNA load, log_10_ viral particles/mL, mean (SD)	6.8 (1.8)	6.9 (1.5)	6.9 (1.5)	6.9 (1.6)
Influenza type, No. (%)				
A	281 (87.3)	263 (87.4)	130 (76.9)	399 (75.9)
B	38 (11.8)	34 (11.3)	37 (21.9)	124 (23.6)
A + B	3 (0.9)	4 (1.3)	2 (1.2)	3 (0.6)
Influenza A subtype, No. (%)				
A/H1N1(2009)	38 (11.8)	23 (7.6)	57 (33.7)	159 (30.2)
A/H3N2	237 (73.6)	235 (78.1)	67 (39.6)	224 (42.6)
Missing or negative subtyping, including B, A + B	45 (14.0)	43 (14.3)	45 (26.6)	143 (27.2)

The ITTI population comprised participants with RT-PCR–confirmed influenza virus infection.

Abbreviations: ITTI, intent-to-treat infected; RT-PCR, reverse transcriptase polymerase chain reaction; SD, standard deviation; TCID_50_, median tissue culture infectious dose.

### Favipiravir Plasma Concentrations

**Figure 1.**
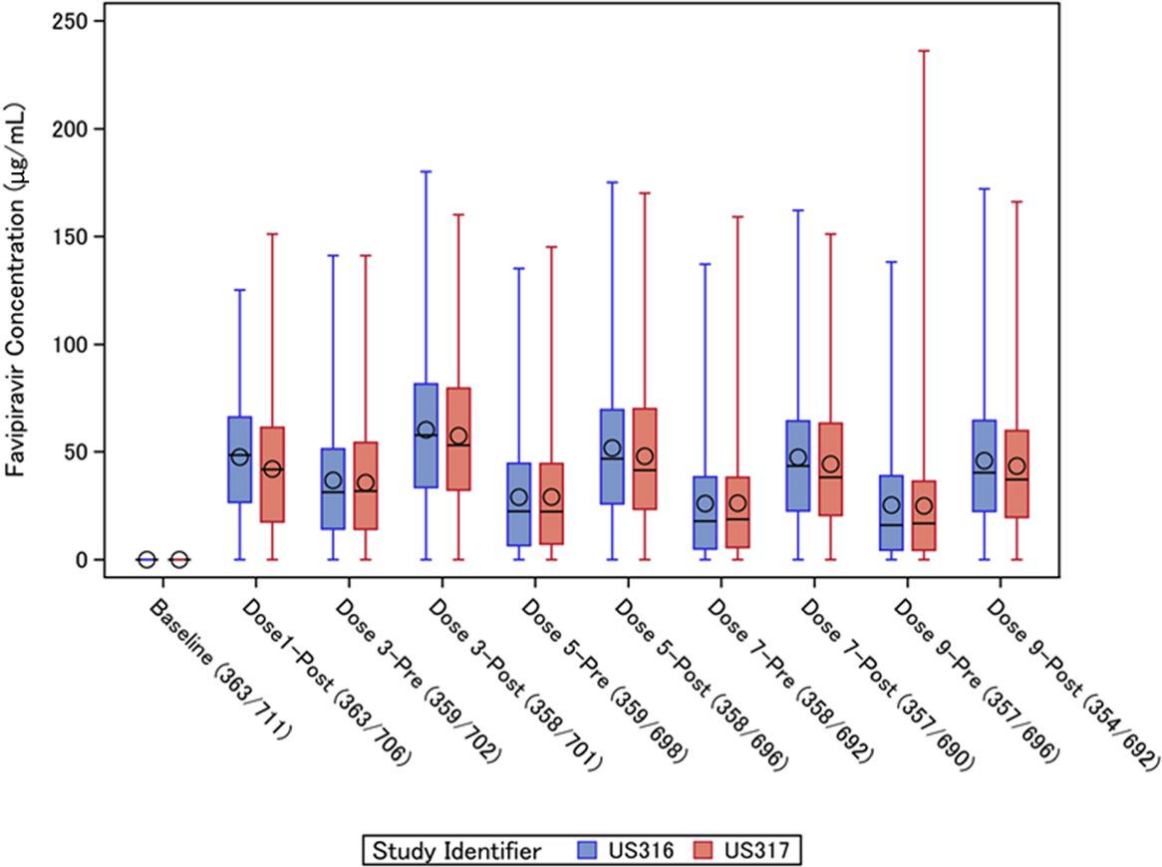
Plasma concentrations of favipiravir before and after dosing in US316 and US317 trials. The lines within the boxes represent the median concentration, the circles represent the mean, the boxes represent the interquartile range, and vertical lines represent the minimum-maximum concentrations. The numbers of samples available for analysis in each of the trials (US316/US317) are listed under the horizontal axis.

In US316 the loading dose of 1800 mg favipiravir resulted in a mean C_max_ of 47.5 (SD, 27.2) μg/mL at 1 hour after the initial dose. Prior to the third dose (800 mg) on study day 2, the mean favipiravir C_min_ was 36.7 (SD, 34.9) μg/mL, and subsequent average C_min_ values exceeded the 20 µg/mL threshold (Figure 1). However, very wide interindividual variations were found for both C_max_ (3.5 to 180 μg/mL) and C_min_ (0.0 to 117 μg/mL) during favipiravir administration.

The mean C_min_ and C_max_ concentrations were highest before and after the third dose on day 2, respectively, and gradually declined at subsequent time points, so that many participants failed to reach the C_min_ target of 20 µg/mL (Figure 1). Similar patterns were observed in US317 (Figure 1). Among evaluable participants, 127 (43.2%) in US316 and 209 (41.0%) in US317 had average C_min_ values <20 µg/mL. In addition, 30 (10.3%) in US316 and 60 (11.6%) in US317 had day 1 C_max_ values below this threshold.

### T-705M1 Plasma Concentrations

**Figure 2.**
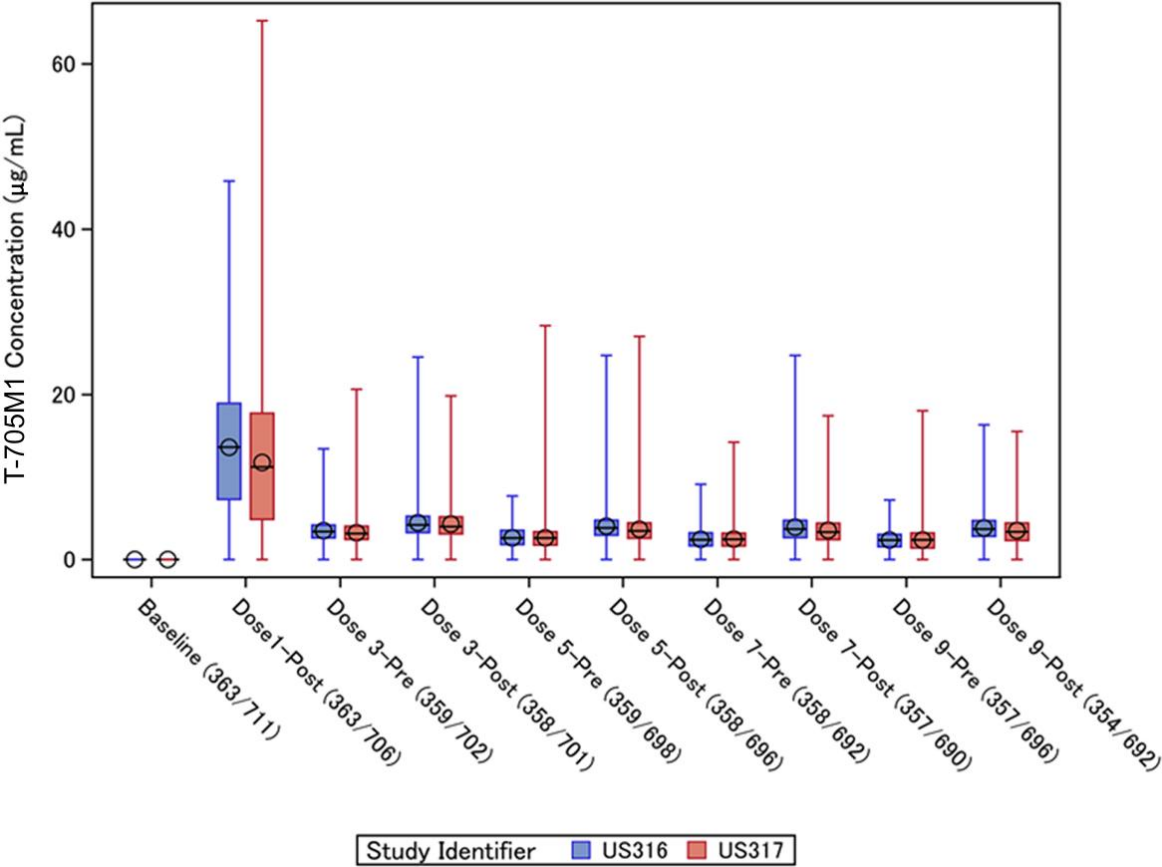
Plasma concentrations of T-705M1 before and after dosing in US316 and US317 trials. The lines within the boxes represent the median concentration, the circles represent the mean, the boxes represent the interquartile range, and vertical lines represent the minimum-maximum concentrations. The numbers of samples available for analysis in each of the trials are listed under the horizontal axis.

The T-705M1 concentrations (Figure 2) were highest after the first loading dose (mean 13.5 µg/mL) and then decreased to an average of approximately 3–4 µg/mL. The T-705M1 concentrations also showed wide interindividual variability. Similar patterns were observed in US317.

**Figure 3.**
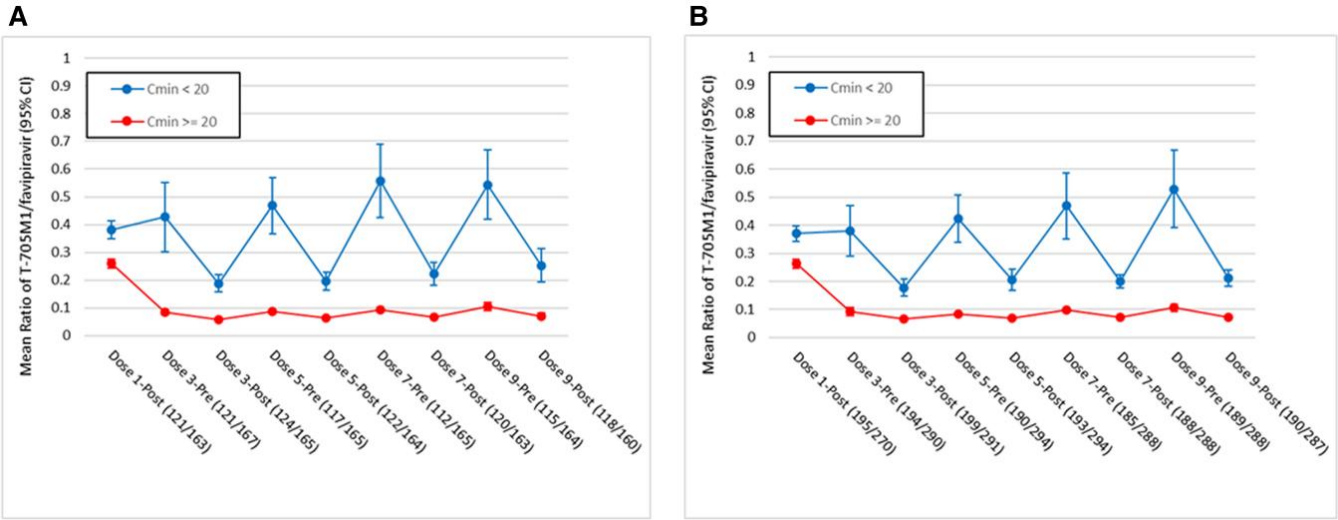
Ratios of plasma concentrations of T-705M1 to favipiravir before and after dosing in US316 (*A*) and US317 (*B*) trials. The lines within the boxes represent the median concentration, the circles represent the mean, the boxes represent the interquartile range, and vertical lines represent the minimum-maximum concentrations. Abbreviations: CI, confidence interval; C_min_, minimum concentration. The numbers of samples available for analysis in each of the trials are listed under the horizontal axis.

Because differences in favipiravir concentrations might relate to the extent of metabolism, we determined the ratios of plasma concentrations of T-705M1 to favipiravir for each individual at each sampling time point (Figure 3 and Supplementary Tables 1 and 2). Of note, in both trials, the ratios of T-705M1 to favipiravir were consistently higher in favipiravir recipients who had average C_min_ values <20 µg/mL compared to those with average C_min_ ≥20 µg/mL across the 5 days of dosing. Among favipiravir recipients who failed to reach C_max_ of 20 µg/mL postdose, the ratios of T-7501M1/favipiravir were over 2-fold higher than those observed in participants with C_max_ ≥20 µg/mL in both US316 (least square mean, 0.39 [95% CI, .36–.42] vs 0.14 [95% CI, .13–.15]) and US317 (least square mean, 0.35 [95% CI, .33–.37] vs 0.14 [95% CI, .13–.14]).

### Relationship Between Favipiravir Exposures and Antiviral Efficacy

As shown in Table 2, significantly larger reductions in infectious virus titers were found in the favipiravir recipients compared to placebo at early time points but not at day 5. Larger reductions were seen in those with C_min_ values reaching the average C_min_>20 µg/mL threshold on days 2, 3, and 5 compared to favipiravir recipients who did not (Table 2). In both trials approximate 0.3–0.4 log_10_ TCID_50_/mL greater reductions were evident by study day 2 in those with favipiravir C_min_ ≥20 µg/mL compared to those with lower concentrations.

**Table 2.** Relationship Between Achieving Minimal Plasma Favipiravir Concentration of 20 µg/mL and Reductions in Infectious Virus Titers From Baseline During Favipiravir or Placebo Treatment

Study	Treatment	Change in Mean (95% CI) Infectious Virus Titer from Baseline, log_10_ TCID_50_/mL					
		Day 1–2, C_min_ < 20 µg/mL	Day 1–2, C_min_ ≥ 20 µg/mL	Day 1–3, C_min_ < 20 µg/mL	Day 1–3, C_min_ ≥ 20 µg/mL	Day 1–5, C_min_ < 20 µg/mL	Day 1–5, C_min_ ≥ 20 µg/mL
US316	Favipiravir	−1.04 (−1.37 to −0.71) (n = 94)*	−1.43 (−1.66 to −1.19) (n = 199)^*,**^	−1.84 (−2.19 to −1.49) (n = 109)*	−2.24 (−2.48 to −1.99) (n = 182)*, **	−2.37 (−2.68 to −2.06) (n = 124)	−2.45 (−2.71 to −2.19) (n = 165)
	Placebo	−0.75 (−0.94 to −0.56) (n = 314)		−1.63 (−1.85 to −1.41) (n = 311)		−2.46 (−2.67 to −2.27) (n = 306)	
US317	Favipiravir	−1.45 (−1.74 to −1.17) (n = 165)*	−1.80 (−1.98 to −1.61) (n = 340)*, **	−2.43 (−2.74 to −2.11) (n = 178)*	−2.73 (−2.93 to −2.53) (n = 326)*	−3.08 (−3.34 to −2.82) (n = 202)	−2.93 (−3.14 to −2.73) (n = 295)
	Placebo	−1.10 (−1.36 to −0.84) (n = 161)		−1.90 (−2.21 to −1.59) (n = 162)		−2.60 (−2.93 to −2.27) (n = 160)	

See Table 1 for baseline infectious virus titers. The proportion of participants with C_min_ < or ≥20 µg/mL on the specific follow-up day 2, 3, or 5 are indicated; 95% confidence intervals are provided in parenthesis for the mean values listed. For analyses of TCID_50_, values below the LLOQ were treated as half the LLOQ.

Abbreviations: CI, confidence interval; C_min_, minimum concentration; LLOQ, lower limit of quantification; TCID_50_, median tissue culture infectious dose.

^*^Indicates the change in TCID_50_ between the C_min_ < 20 µg/mL versus placebo or C_min_> 20 µg/mL versus placebo is statistically significant at α level of .05.

^**^Indicates the change in TCID_50_ between the C_min_ < 20 µg/mL versus C_min_ ≥20 µg/mL is statistically significant, at α level of .05. In US316, the reduction in the mean titer was greater in participants with C_min_ ≥20 ug/ml over time, and the change was statistically significant on day 2 and day 3. In US317, similar patterns in the magnitude of reductions between the favipiravir subgroups were observed.

In both trials, mean TCID_50_ AUC values (expressed in TCID_50_ × h/mL) were higher in placebo recipients compared to those with average favipiravir C_min_ <20 µg/mL (US316, 144 [95% CI, 134–154] vs 109 [95% CI, 96–122], *P* = .0001; US317, 153 [95% CI, 137–168] vs 125 [95% CI, 115–135], *P* = .0025). The mean TCID_50_ AUC values were decreased to greater degrees in those with average C_min_ ≥20 µg/mL in both US316 (100 [95% CI, 91–108], *P* < .0001, compared to placebo) and US317 (109 [95% CI, 102–115], *P* < .0001). The AUC values in those with average C_min_ ≥20 µg/mL were numerically lower compared to those with average C_min_ <20 µg/mL in US316 (*P* = .22) and significantly lower in the US317 (*P* = .007).

Survival analysis found that the time to undetectable infectious virus was about 24 hours shorter in the favipiravir groups compared to placebo in both trials [16]. However, the median time to undetectable infectious virus was not different between the favipiravir subgroups with or without C_min_ ≥20 µg/mL in either US316 (median, 47.5 vs 47.5 hours) or US317 (47.5 vs 47.8 hours).

### Relationship Between Favipiravir Exposures and Illness Alleviation

Our earlier post hoc analysis of US316 found that favipiravir recipients who had average C_min_ ≥20 µg/mL had a significantly shorter (15.3 hour) difference in median time to illness alleviation compared to placebo [16] (Table 3). In US317 a nonsignificant reduction of 11.0 hours was observed in those with average C_min_ ≥ 20 µg/mL compared to placebo (Table 3). In both trials favipiravir recipients with average C_min_ <20 µg/mL had minimal differences (2.9–3.4 hours) in the median time to alleviation compared to placebo recipients.

**Table 3.** Relationship Between Mean Minimal Plasma Favipiravir Concentration of 20 µg/mL and Primary Clinical End Point of Time to Alleviation of Acute Influenza Illness

Study	Mean Favipiravir C_min_, µg/mL	No. Evaluable Participants	Time To Illness Alleviation, h, Median (95% CI)	*P* Value, Favipiravir Subgroup vs Placebo
US316	C_min_ ≥ 20	167	83.3 (71.8–95.5)	.003
US316	C_min_ < 20	134	95.7 (77.1–101.1)	.081
US316	Placebo	322	98.6 (94.6–107.1)	
US317	C_min_ ≥ 20	301	72.9 (71.7–82.0)	.157
US317	C_min_ < 20	224	80.5 (76.0–95.6)	.778
US317	Placebo	169	83.9 (76.0–95.5)	

Abbreviations: CI, confidence interval; C_min_, minimum concentration.

### Effect of Body Weight on Favipiravir Exposure and Antiviral Efficacy

The proportions of favipiravir recipients with average C_min_ <20 µg/mL were much higher in those weighing ≥80 kg compared to those weighing less in both US316 (61.5% vs 24.7%) and US317 (63.8% vs 19.5%) (Table 4). In addition, the day 1 postdose C_max_ was lower in those weighing ≥80 kg compared to those weighing less in both US316 (mean, 39 µg/mL [95% CI, 36–42] vs 63 [95% CI, 59–68]; *P* < .001) and in US317 (mean, 37 [95% CI, 34–9] vs 67 [95% CI, 52–82]; *P* = .001). Among the participants who failed to reach a C_max_ ≥20 µg/mL after the initial loading dose on day 1, 22 (73%) of 30 in US316 and 46 (77%) of 60 in US317 weighed 80 kg or more.

**Table 4.** Participant Body Weight and Effects on Favipiravir Concentrations and Antiviral Efficacy

Body Weight	No. (%) With Average Favipiravir C_min_ <20 µg/mL	Viral Titer AUC, Mean (95% CI)	No. (%) With Average Favipiravir C_min_ ≥20 µg/mL	Viral Titer AUC, Mean (95% CI)
US316 trial				
Weight <80 kg	36 (24.7)	102.5 (82.4–122.6)	110 (75.3)	104.1 (93.1–115.2)
Weight ≥80 kg	91 (61.5)*	111.6 (95.1–128.0)	57 (38.5)	91.0 (77.2–104.9)
US317 trial				
Weight <80 kg	51 (19.5)	114.2 (95.7–132.6)	210 (80.5)	112.5 (104.3–120.6)
Weight ≥80 kg	157 (63.8)*	128.5 (116.8–140.3)	90 (36.2)	101.0 (88.1–113.8)

Abbreviations: AUC, area under the curve; CI, confidence interval; C_min_, minimum concentration.

^*^ *P* value that is less than or equal to .05.

Favipiravir recipients weighing ≥80 kg and having average C_min_ <20 µg/mL tended to have reduced antiviral efficacy, based on viral titer AUCs, compared to those weighing less and having average C_min_ <20 µg/mL, but this pattern was not observed in those weighing ≥80 kg and having average C_min_ ≥20 µg/mL (Table 4). In US316, the ratio of T-705M1 to favipiravir after the dose on day 1 was higher in those weighing ≥80 kg who failed to reach the 20 µg/mL threshold compared to those weighing ≥ 80 kg who did (least square mean ratio, 0.39 [95% CI, .36–.43] vs 0.16 [95% CI, .15–.18]; *P* < .05). Similar findings were found in US317 (least square mean ratio, 0.35 [95% CI, .32–.37] vs 0.16 [95% CI, .15–.17]; *P* < .05).

## DISCUSSION

These post hoc analyses documented consistent PK patterns across the 2 phase 3 favipiravir treatment trials, which corresponded to their similar magnitudes of antiviral effects, but did not identify PK factors explaining the differences observed in influenza illness alleviation [16]. It is possible that the higher baseline infectious virus titers found in favipiravir participants in US317 compared to US316 were contributory. We found evidence for greater antiviral efficacy early in those with higher favipiravir exposure based on an average C_min_ ≥20 µg/mL, although participants with lower average C_min_ also showed reductions in infectious virus compared to placebo. Higher favipiravir exposure was associated with greater reductions in infectious virus area-under-the-curve values compared to those not reaching the average C_min_ of 20 µg/mL. No important effect was seen on the duration of infectious virus detection between the groups, possibly because many participants had nondetectable viral titers after the first few days.

Important findings of these trials were the wide ranges in favipiravir plasma concentrations and overall exposures in ambulatory adults with influenza. We found that many participants had low favipiravir C_min_ values despite initial loading doses and that the numbers of participants with low C_min_ tended to increase over the 5 days of dosing. This confirms other studies reporting considerable interindividual variation in observed plasma concentrations in acutely ill patients [15, 17, 18]. The reasons for these differences are not fully understood, but our findings provide evidence that both favipiravir metabolism differences and higher body weight impacted plasma concentrations of favipiravir and its associated antiviral effects. Among those with body weight <80 kg, 23%–27% did not reach day 1 C_max_ concentration ≥20 µg/mL and 20%–25% did not achieve an average C_min_ ≥20 µg/mL, which suggests that differences in oral bioavailability may also have been a factor.

PK studies in cynomolgus macaques showed the conversion of favipiravir into T-705 M1 is almost instantaneous after the first dose, but is reduced in subsequent doses (Robert Lenk, personal communication). In seriously ill patients hospitalized with influenza, Ebola, and recently coronavirus disease 2019 (COVID-19) who were given oral favipiravir, plasma concentrations were much lower than predicted and greater declines in concentrations over time despite continued dosing have been reported [4, 19, 20]. It is likely that intrinsic variability in AO activity is an important contributor to the observed variations in favipiravir concentrations [21]. Perhaps acute inflammation related to infection elevates AO activity in affected tissues and exaggerates such variability. In our trials, the plasma concentrations of the principal favipiravir metabolite, T-705-M1, also showed considerable variability and dropped quickly after peaking following the day 1 loading doses. Of note, the plasma T-705M1 to favipiravir ratios were significantly higher in the favipiravir recipients having inadequate favipiravir plasma concentrations throughout the dosing period, consistent with greater favipiravir metabolism contributing to the lower plasma concentrations in our trials and supporting the hypothesis that differences in AO metabolic activity due to genetic factors or inflammation at least partially explain the high interindividual variations we observed in favipiravir concentrations.

The initial analysis of the primary clinical end point found that those weighing 80 kg or more had no benefit in regard to illness alleviation from favipiravir treatment in either trial [16]. We also found that those weighing ≥80 kg had lower favipiravir exposures based on greater proportions with average C_min_ ≤20 µg/mL. Also, approximately three-quarters of favipiravir participants failing to reach an initial postdosing C_max_ of 20 µg/mL weighed ≥80 kg. The lower exposures were associated with lesser antiviral efficacy. Among those weighing ≥80 kg, the T-750M1 to favipiravir ratios were higher in those failing to achieve a postdose day 1 C_max_ of 20 µg/mL, indicating greater levels of favipiravir metabolism in the former group. These observations suggest that higher, weight-adjusted oral favipiravir dose regimens should be studied in influenza outpatients, especially in those weighing ≥80 kg. Of note, much higher oral dose regimens (up to 6000 mg on day 1 followed by 2400 mg on day 2–9 in divided doses) have been used in treating Ebola patients without obvious safety problems [3].

Because of the unpredictability of oral favipiravir exposure, especially in severely ill patients, the development of an intravenous formulation to provide reliable delivery would be advisable for study in hospitalized patients. In this regard, a study of 7 critically ill COVID-19 patients who were administered suspensions of favipiravir tablets (1600 mg of FPV twice daily on day 1, followed by 600 mg twice daily) through a nasogastric tube found that the favipiravir C_min_ concentrations (after 8–12 hours) of most samples were below the LLOQ (1 μg/mL) [17], An intravenous formulation would also help address questions regarding oral bioavailability and metabolism to help understand the mechanisms contributing to inadequate favipiravir exposure in such patients. Of note, a dose-ranging phase 1 trial of intravenous favipiravir is currently being conducted in COVID-19 by investigators in the AGILE consortium (ClinicalTrials.gov identifier, NCT04746183).

Compared to influenza viruses, most RNA viruses are less susceptible to favipiravir in vitro [1, 2, 22, 23], and require up to 10-fold higher weight-based doses in murine models to demonstrate antiviral efficacy [24–26] compared to influenza [1, 2]. Our results indicate that much higher dose regimens than the one used in our influenza trials, such as employed in Ebola studies [3, 19], would be required to treat many other viral infections. In this regard, coronaviruses are much less susceptible to favipiravir in vitro than influenza, and high parenteral (intravenous followed by subcutaneous doses) that gave C_max_ of approximately 300 µg/mL and C_min_ of approximately 75–80 µg/mL failed to exert anti–SARS-CoV-2 effects in a nonhuman primate model [27]. Despite some claims that dose regimens similar to the one we tested in influenza have benefited COVID-19 patients [28, 29], placebo-controlled trials in outpatients [30–32] and smaller trials in hospitalized patients, one placebo-controlled [33] and the other randomized by timing of treatment initiation [6], did not demonstrate significant antiviral effects or clinical benefit.

Our analyses have several limitations. The sparse extent of sampling due to the large numbers of participants limited consideration of other PK variables. We could not assess oral bioavailability, and no data were obtained on the respiratory tract distribution of favipiravir or, importantly, intracellular levels of favipiravir-RTP. Inter-individual differences in favipiravir-RTP's intracellular concentrations and metabolism may be especially relevant with respect to antiviral effectiveness. Also, we did not take into account favipiravir's plasma protein binding (approximately 54%), and the considerable variability we observed in upper respiratory tract viral titers, times to symptom resolution, and favipiravir exposures precluded a determination of optimal exposure thresholds in uncomplicated influenza. However, our findings demonstrate the importance of achieving sufficient plasma favipiravir exposures in treating acutely ill patients and support further exploration of alternative dosing regimens.

In summary, we found wide interindividual variability in favipiravir plasma concentrations in 2 large cohorts of adults treated for acute, uncomplicated influenza. Higher favipiravir levels, as reflected in average plasma C_min_ ≥20 µg/mL, were associated with larger antiviral effects and more rapid illness alleviation compared to placebo and to favipiravir recipients with lower average C_min_ values. Lower favipiravir concentrations were associated with both more rapid metabolism and body weight of 80 kg or greater. These findings have important implications for dose selection in future clinical studies of favipiravir in influenza and other RNA virus infections.

## Supplementary Data

Supplementary materials are available at *The Journal of Infectious Diseases* online. Consisting of data provided by the authors to benefit the reader, the posted materials are not copyedited and are the sole responsibility of the authors, so questions or comments should be addressed to the corresponding author.

## Supplementary Material

jiad409_Supplementary_Data

## Notes

***Financial support*.** This work was supported by the United States Department of Defense (US316 and US317 trials contract number HDTRA1-12-C-0031).
