# Supplementary material for: Oral Favipiravir Exposure and Pharmacodynamic Effects in Adult Outpatients With Acute Influenza
Source: J Infect Dis. 2023 Sep 22;230(2):e395–404. doi: 10.1093/infdis/jiad409 (PMC11326817; doi:10.1093/infdis/jiad409)
Supplement: jiad409_Supplementary_Data [file jiad409_supplementary_data.pdf]

Supplementary Table 1. Ratios of T-705M1/Favipiravir Between Cmin <20 vs Cmin ≥20 ug/ml in ITT-Infected Population (US316)

Obs	Group	Analysis Timepoint	Analysis Visit	n	Mean	Standard Deviation	Upper 95% CI	Lower 95% CI
1	Cmin < 20	Post-Dose	Day 1	121	0.38107	0.17995	0.41346	0.34868
2	Cmin < 20	Post-Dose	Day 2	124	0.18872	0.17646	0.22008	0.15735
3	Cmin < 20	Post-Dose	Day 3	122	0.19529	0.18171	0.22786	0.16272
4	Cmin < 20	Post-Dose	Day 4	120	0.22234	0.22440	0.26290	0.18178
5	Cmin < 20	Post-Dose	Day 5	118	0.25290	0.33172	0.31338	0.19242
6	Cmin < 20	Pre-Dose	Day 2	121	0.42777	0.69393	0.55267	0.30287
7	Cmin < 20	Pre-Dose	Day 3	117	0.46786	0.55988	0.57038	0.36534
8	Cmin < 20	Pre-Dose	Day 4	112	0.55671	0.70894	0.68946	0.42397
9	Cmin < 20	Pre-Dose	Day 5	115	0.54325	0.67794	0.66849	0.41802
10	Cmin >=20	Post-Dose	Day 1	163	0.25977	0.10844	0.27654	0.24300
11	Cmin >=20	Post-Dose	Day 2	165	0.05963	0.02968	0.06419	0.05506
12	Cmin >=20	Post-Dose	Day 3	164	0.06354	0.03783	0.06938	0.05771
13	Cmin >=20	Post-Dose	Day 4	163	0.06733	0.03679	0.07302	0.06164
14	Cmin >=20	Post-Dose	Day 5	160	0.07137	0.06204	0.08106	0.06169
15	Cmin >=20	Pre-Dose	Day 2	167	0.08539	0.05330	0.09353	0.07725
16	Cmin >=20	Pre-Dose	Day 3	165	0.08682	0.04554	0.09382	0.07982
17	Cmin >=20	Pre-Dose	Day 4	165	0.09392	0.05616	0.10256	0.08529
18	Cmin >=20	Pre-Dose	Day 5	164	0.10525	0.08932	0.11902	0.09148
19	NA	Post-Dose	Day 1	7	0.32937	0.13741	0.45645	0.20229

Supplementary Table 2. Ratios of T-705M1/Favipiravir Between Cmin <20 vs Cmin ≥20 ug/ml in ITT-Infected Population (US317)

Obs	Flag	Analysis Timepoint	Analysis Visit	n	Mean	Standard Deviation	Upper 95% CI	Lower 95% CI
1	Cmin < 20	Post-Dose	Day 1	195	0.37005	0.18983	0.39686	0.34324
2	Cmin < 20	Post-Dose	Day 2	199	0.17792	0.22444	0.20929	0.14654
3	Cmin < 20	Post-Dose	Day 3	193	0.20499	0.26946	0.24325	0.16673
4	Cmin < 20	Post-Dose	Day 4	188	0.19962	0.16519	0.22338	0.17585
5	Cmin < 20	Post-Dose	Day 5	190	0.21091	0.20565	0.24034	0.18148
6	Cmin < 20	Pre-Dose	Day 2	194	0.37980	0.63505	0.46973	0.28988
7	Cmin < 20	Pre-Dose	Day 3	190	0.42335	0.59522	0.50853	0.33817
8	Cmin < 20	Pre-Dose	Day 4	185	0.46894	0.81030	0.58648	0.35140
9	Cmin < 20	Pre-Dose	Day 5	189	0.52919	0.96955	0.66831	0.39007
10	Cmin >=20	Post-Dose	Day 1	270	0.26269	0.12989	0.27826	0.24713
11	Cmin >=20	Post-Dose	Day 2	291	0.06585	0.05020	0.07164	0.06006
12	Cmin >=20	Post-Dose	Day 3	294	0.06925	0.04946	0.07493	0.06357
13	Cmin >=20	Post-Dose	Day 4	288	0.07298	0.04836	0.07859	0.06737
14	Cmin >=20	Post-Dose	Day 5	287	0.07275	0.04743	0.07826	0.06724
15	Cmin >=20	Pre-Dose	Day 2	290	0.09182	0.12158	0.10587	0.07776
16	Cmin >=20	Pre-Dose	Day 3	294	0.08491	0.04895	0.09053	0.07929
17	Cmin >=20	Pre-Dose	Day 4	288	0.09724	0.06889	0.10523	0.08925
18	Cmin >=20	Pre-Dose	Day 5	288	0.10596	0.10950	0.11866	0.09326
19	NA	Post-Dose	Day 1	15	0.31062	0.15029	0.39385	0.22740
